# 

**Published:** 1871-04

**Authors:** 


### CONTENTS:

ORIGINAL COMMUNICATIONS:

ARTICLE I. Matter: Living and Dead. By I. N. Danforth, M. D.. 193

ART. II. Report of Vivisections, etc., performed by Prof. J. W. Freer, M. D., before the Alumni of Rush Medical College, Chicago. By F. L. Wadsworth, M. D...... 200

ART. III. Criticism on a Recently Reported Case—Scarlatinal Sequela, treated with Veratrum Viride. By W. H. Boyce, M. D............. 208

ART. IV. Pepsine and its Incompatibles. By I. S. Hawley, M. D... 212

ART. V. Case of Fracture of the Anatomical Neck of the Humerus, and Recovery. By E. Duffield, M. D.................................. 214

ART. VI. Case of Transplantation of Skin, at Cook Co. Hospital. By E. Powell, M. D.................. 219

ART. VII. Opium in Metro-Peritonitis. Review of a Criticism. By W. L. Johnson, M. D.......... 220

SELECTIONS.......................... 225

EDITOR'S BOOK TABLE.................. 233

EDITORIAL........................... 248

NOTICES.............................. 255

